# Fatigue risk assessment of a Helicopter Emergency Medical Service crew working a 24/7 shift pattern: results of a prospective service evaluation

**DOI:** 10.1186/s13049-023-01143-4

**Published:** 2023-11-03

**Authors:** C. Rose, E. ter Avest, R. M. Lyon

**Affiliations:** 1 Kent, Surrey and Sussex Air Ambulance Trust, Redhill Airfield, Redhill, Surrey, RH1 5YP UK; 2 Department of Emergency Medicine, University Hospital Groningen, Groningen, The Netherlands; 3 School of Health Sciences, University of Surrey, Guildford, UK

**Keywords:** Fatigue, Performance, Workload, HEMS, Air Ambulance

## Abstract

**Background:**

The work of Helicopter Emergency Medical Services (HEMS) teams crosses the boundaries of several high-risk occupations including medicine, aviation, and transport. Working conditions can be challenging and operational demands requires a 24-h rota, resulting in disruption of the normal circadian rhythm. HEMS crews are therefore prone to both mental and physical fatigue. As fatigue in medical providers is linked to poor cognitive performance, degradation of psychomotor skills and error, this study aimed to explore the existence of predictable patterns of crew-fatigue in a HEMS service.

**Methods:**

HEMS medical crew members working a 3-on 3-off forward rotating rota with a 5-week shift cycle were asked to do psychomotor vigilance tests (PVT) as an objective measure of fatigue. PVT testing was undertaken at the start, mid- and at the end of every shift during a full 5-week shift cycle. In addition, they were asked to score subjective tiredness with the Samn-Perelli Fatigue Scale (SPFS), and to keep a Transport Fatigue Assessment shift log, wherein they noted shift characteristics potentially related to fatigue. Primary outcome of interest was defined as the change in PVT and SPFS scores over time.

**Results:**

Mean baseline resting PVT in milliseconds at the start of the study period was 427 [390–464]. There was an overall trend towards higher PVT-scores with shift progression mean [95% CI] PVT at the start of shifts 447 [433–460]; halfway through the shift 452 [440–463]; end of the shift 459 [444–475], *p* = 0.10), whereas SPFS scores remained constant. Within a 5 week forward-rotating cycle, an overall trend towards a gradual increase in both average PVT (from 436 [238–454] to 460 [371–527, *p* = 0.68] ms;) and SPFS (from 2.9 [2.6–3.2] to 3.6 [3.1–4.0], p = 0.38) was observed, although significant interindividual variation was present. Reported SPFS scores ≥ 4 (moderate fatigue) were mainly related to workload (number of jobs) and transport mode (car-based shifts).

**Conclusion:**

An overall trend towards a decline in psychomotor vigilance and an increase in self-reported tiredness was found for HEMS crew over a 5-week shift cycle. Using a bespoke predictive fatigue tool on a day-to-day basis could increase fatigue awareness and provide a framework to which relevant mitigating options can be applied.

## Background

Fatigue is a subjective symptom generally described as a feeling of tiredness or exhaustion [1]. Fatigue in medical providers is linked with poor cognitive performance, degradation of psychomotor skills and ultimately clinical error [2, 3] which can compromise patient safety. This can arise from excessive working time or poorly designed shift patterns [4]. Problems occur when workload outweighs the opportunity for rest and recovery. If left unchecked, fatigue can become a risk to service users and potentially endanger the providers well-being [5].

HEMS crews are generally comprised of a highly skilled medical crew and one- or two pilots. Medical crew members are required to maintain a high level of performance around the clock [6] whilst managing a complex and unpredictable workload of critically ill patients [7]. They deliver enhanced care such as emergency anaesthesia, surgical interventions and blood product administration. In addition to that, they share responsibility for the safety of the flight and the operation of a blue light response vehicle. Fatigue increases the probability of poorer task management [3], whilst the margin for error is already slim.

So far, knowledge of the impact of fatigue on the performance of HEMS teams is limited. Several small feasibility studies reported on the influence of fatigue on readiness to perform critical tasks [3, 8, 9], whereas other studies looked at the impact of shift length on fatigue [10–12]. However, significant knowledge gaps still exist: only one study [13] looks at the effect of cumulative fatigue in HEMS crews over multiple shifts, and none of the previous studies related objective measures of fatigue to a subjective feeling of tiredness reported by crews.

Therefore, in the present study, we aim to explore if a reproducible pattern of fatigue exists among a HEMS 24/7 rota by investigating both objective and subjective fatigue with previously validated instruments.

## Methods

## Study design

A prospective observational study of HEMS crew members working a 3-on 3-off forward rotating rota was performed to examine the relation between fatigue and shift length, shift type (early, day, late, night or relief) and cumulative number of shifts within a 5-week rota cycle. Fatigue was measured objectively before, during and after each shift by reaction time-task monitoring for changes in behavioural alertness [14] and subjectively by crew self-reporting fatigue levels using the Samn–Perelli Fatigue Scale (SPFS) [15]. In addition to this, crews kept a shift log using the Transport Fatigue Assessment (TFA) [16] throughout the entire study period to relate measured and reported fatigue to shift characteristics and individual circumstances.

### Study setting

The study was performed amongst medical crew (doctors and paramedics) of Air Ambulance Kent, Surrey and Sussex (AAKSS). AAKSS HEMS covers three counties in the southeast of England, a region of 7200 km^2^ with a resident population of 4.5 million, and a transient population of 8 million. Two doctor-paramedic teams respond 24/7 in either a helicopter or rapid response vehicle from one operational base, attending approximately 2000 patients per year. The service operates four crews on two aircraft over a 24-h period. One team operates from 0700 to 1900 (dayshift) followed by a crew from 1900 to 0700 (nightshift). The second team operates from 0600 to 1500 (early shift) followed by a team from 1500 to 0000 (late shift). Periods of the early and late shift are operated on the response car. Response vehicles are also used during time the aircraft cannot fly either due to weather or maintenance. Each run of shifts is over three consecutive days. Rest days follow a three on, three off, three on, two off pattern due to rostered clinical governance days. The service has a policy in place to prevent fatigue amongst crews, including a mandatory minimum 11-h rest between shifts and the allowance of controlled rest during shifts.

The study period took place between 7 June and 2 August 2021. All participants started the study period with a minimum of 24 h rest prior to their first shift to collect a baseline rested data set. Each participant progressed through the five-week cycle following the same forward rotating pattern of day/early/late/night/relief shifts dependent on their starting point in the cycle. Relief shifts are a run of three of any shift type, or a mix of shift type, with the potential to disrupt the natural forward rotating pattern of the rota.

### Study population

Recruitment of medical crew occurred over a 1-week period from 1/6/21 to 6/6/21. Selection of participants was by means of non-probability convenience sampling,, consisting of both doctors and paramedics, male and female. A pre-defined sample size of 8 participants provided a manageable cross-section of the study population, representing almost two thirds of the eligible full-time employees. These were selected from a limited pool of those available to work full-time during a complete cycle of the 5-week rota. All selected individuals received information about the study beforehand and were given 48 h to decide whether to participate in the study. Written informed consent for participation was obtained from all participating subjects. Study data was only accessible to the study team, not to the organisation.

### Data acquisition

A dedicated PVT Research App [17] was pre-loaded to the relevant shifts mobile phone, each of which used the same software and hardware combinations. The application was used with all windows shut down and WiFi temporarily off to reduce test variability by controlling the latency of systems running in the background. The app provided a 3-min test with interstimulus intervals (ISI) between 1 and 4 s: Study subjects had to tap the screen (in portrait mode) with the thumb of their dominant hand as soon as a red dot appeared. PVT outcomes were measured by mean response speed in milliseconds (ms). Valid response times were regarded as > 100 ms. Lapses were defined as response times > 355 ms. Pressing the button in anticipation prematurely was considered a false start. If the button was not pushed within 30 s, this was considered a timeout. Mean, minimum and maximum response times, false starts, timeouts and lapses were recorded in the app. The PVT was recorded by each participant at the start, midpoint and end of each shift. Average baseline rested state was calculated from the eight individual participant response times of the second PVT test on day one. Although no learning effect is associated with the PVT [18], the second score was chosen due to increased familiarity with the application and study procedures.

At the same moment, SPFS scores were reported in whole numbers. 1 = ‘fully alert, wide awake’; 2 = ‘very lively, responsive but not at peak’; 3 = ‘Okay, somewhat fresh’; 4 = ‘a little tired, less than fresh’; 5 = ‘moderately tired, let down’; 6 = ‘extremely tired, very difficult to concentrate’; and 7 = ‘completely exhausted, unable to function effectively’ [15]. A TFA was kept by all participants, providing a numerical score relating to the participants level of risk based on sleep, shift duration and intensity, and when they last ate or consumed a caffeinated drink, providing a predictive measure of fatigue and anticipated associated performance.

All data of the PVT application was stored locally on the user’s device, downloaded after the shift and shared with the study lead. A personalised anonymised digital data collection folder was made available for each participant to store SPF and TFA data until the end of the study.

### Ethical approval

The service evaluation was granted favourable ethical approval by the SGUL Joint Research and Enterprise Services. The service evaluation was also approved by the Service’s operational directorship and internal Research and Development Team.

### Statistical analysis

PVT and SPFS scores are reported as mean [95% CI] and lapse rates as [%]. Repeated measures ANOVA or non-parametric Friedman’s test (if appropriate) were used to compare PVT scores during the shift, across a run of three shifts and during the five-week shift cycle. Shift type, week number and participant number were entered as between subject factors to investigate interactions with changes in PVT. Lapse rates were compared using Chi-square test. A *p*-value of < 0.05 was considered statistically significant. All analyses were performed using IBM SPSS Statistics for Macintosh, Version 27.

## Results

### Baseline characteristics and data completeness

Five participants were male and 3 were female. Median age was 42 years (range 34–54). 3 doctors and 5 HEMS Specialist paramedics participated. Doctors were from either an Emergency Medicine or Anaesthetics specialty. Experience within the HEMS service ranged from < 1year to 8 years.

For PVT, 263 valid responses (73%) were available for analysis. Missing data were the result of absence (n = 54, 15%) or workload (n = 43, 12%). For SPFS, 266 responses were recorded (73.8%).

### Objective measures of fatigue-PVT

#### PVT during individual shifts

Mean baseline resting PVT at the start of the study period was 427 [390–464] milliseconds (ms) (range 367–490 ms).

Overall mean [95% CI] PVT at the start of any shift during the study period was 447 ms [433–460]. It increased slightly to 452ms [440–463] halfway through the shift, and 459 ms [444–475] at the end of the shift (*p* = 0.10). In comparison to other shift types, nightshifts were the only shifts wherein PVT increased significantly during the course of the shift (Table 1). Lapse rate (as a percentage of the number of recorded PVT’s) was 95.7% at the start of the shift, 94.6% mid shift and 96.0% at the end of the shift (*p* = 0.89).

**Table 1:** Mean [95% CI] PVT in milliseconds before, during and after HEMS shifts stratified by shift type

	Start shift	Mid shift	End shift	*P*
*Shift type*				
Early	457 [430–486]	454 [419–488]	453 [428–478]	0.70
Day	461 [420–502]	469 [436–502]	467 [415–519]	0.86
Late	441 [419–462]	440 [424–454]	452 [428–478]	0.46
Night	420 [380–460]	446 [417–474]	454 [418–489]	0.03
Relief	464 [428–500]	455 [414–496]	454 [417–490]	0.92

#### PVT during a run of shifts

During a run of 3 consecutive shifts there was no change in performance as measured by PVT: Mean [95%CI] PVT was 434 ms [403–467] during the first shift, 424 ms [391–456] during the second shift and 418 ms [383–453] during the third shift, *p* = 0.16. Shift type or week number of the shift cycle did not have a significant interaction with the change in PVT during a run of three shifts (*p* = 0.40 and *p* = 0.07 respectively), but subject number had (*p* = 0.005) indicating a significant variation in PVT change amongst participants in how they coped with the 3 consecutive shifts.

#### PVT during a 5-week shift cycle

**Fig. 1:**
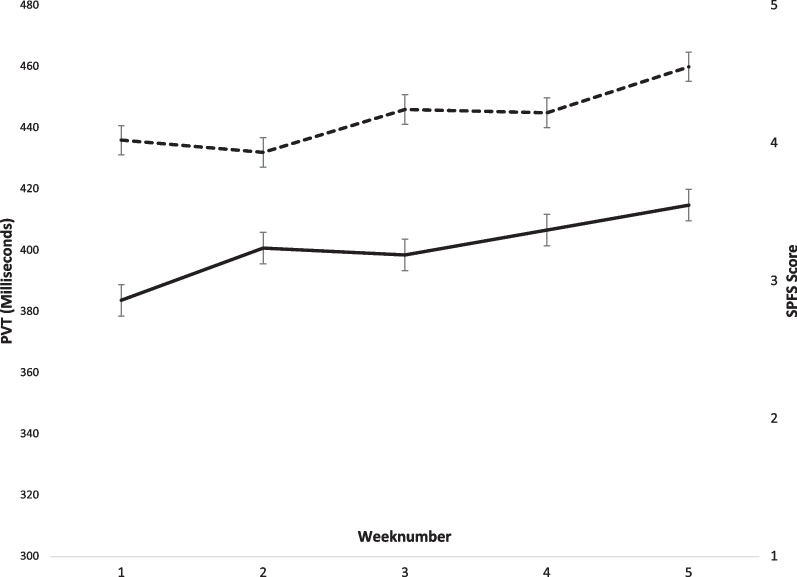
Average PVT- and SPFS scores during a full 5-week shift cycle. PVT, Psychomotor vigilence testing; SPFS, Samn-Perelli Fatigue scale

During a 5-week shift cycle, mean PVT increased gradually in 6/8 subjects, remained constant in one subject, and decreased in 1 subject. Mean PVT increased from on average 436 [404–468] ms in the first week to 460 [410–510] ms in the 5^th^ week (*p* = 0.68, Fig. 1). Lapse rate remained high during this period: 98.3% in week 1, 95.3% in week 5.

### Self-reported tiredness-SPFS

Mean [95% CI] reported SPFS score during the study period was 3.2 [3.1–3.4]. The highest mean SPFS scores were reported during nightshifts (3.5 [2.9–4.0]) and relief shifts (3.5 [3.0–4.0] whereas lowest scores were reported during the (shorter) late shifts 3.0 [2.5–3.6]. No trend was observed for SPFS scores during individuals shifts, or during a row of three shifts. During the full 5-week cycle, the average SPFS score increased slightly but not significantly from 2.9 [2.6–3.2] in week 1, to 3.6 [3.1–4.0] in week 5, *p* = 0.38. (Fig. 1).

Distribution of reported SPFS scores is represented in Table 2. TFA shift log factors associated with a SPFS score of 4 or more (moderate fatigue), were: the number of missions (20%), shifts in response cars (20%), number of stand downs (14%), poor sleep prior to shift (13%) or more than 6 h without sustenance (11%).

**Table 2:** Distribution of SPFS scores recorded during a 5-week shift cycle, (n = 266)

	N (%)
1. Fully alert, wide awake	37(14%)
2. Very lively, responsive, but not at peak	41 15%)
3. Okay, somewhat fresh	69 26%)
4. A little tired, less than fresh	69 26%)
5. Moderately tired, let down	44 17%)
6. Extremely tired, very difficult to concentrate	6 (2%)
7. Completely exhausted, unable to function	0

## Discussion

In the present study it is demonstrated that, despite significant inter-individual variation, a reproducible pattern of fatigue with a decline in psychomotor vigilance and self-reported tiredness occurs throughout a five-week HEMS shift cycle.

Our findings show that it is feasible to monitor crew fatigue with relatively simple tools during day-to-day practice in a HEMS environment, at little extra time expense to the crews. Compliance was high (> 70%, and even higher when lost entries due to absence were disregarded). Using a combination of quantitative and qualitative measures of fatigue, has several advantages, as fatigue is a complex social phenomenon. As fatigue impacts our own self-awareness of performance, sole collection of subjective data potentially compromises internal validity [18]. Further, qualitative data collected about self-reported effects of fatigue in isolation can lack context. Each shift is unique due to the unpredictability of the workload, environmental factors beyond our control and simply how the individual is feeling. Shift logs can help to account for these effects.

In our study, baseline rested state PVT was well above the application rated lapse time of 355 ms. This correlates with previous medical and aviation literature [12, 19, 20] wherein a reduced level of alertness was observed at the start of a run of shifts. This may be explained by a circadian phase delay with later bedtimes or rise times during a preceding run of shifts. Alternatively, higher baseline rested states can be the result of poor recovery between shifts [21]: Shift-workers subjected to chronic sleep restriction over months or years eventually reset their baseline, acclimating to their level of impaired alertness. A low level of exhaustion becomes the norm [22].

Over the course of a single HEMS shift we found that average PVT scores increased slightly, but SPFS scores remained unchanged. It is reasonable to assume that a high level of focus during many hours on shift results in fatigue amongst HEMS crews, as in previous literature an association between fatigue and shift duration has been reported for emergency medical services personnel [23]. This is an important finding, as fatigue impact not only affects mental performance and task management, but also situational awareness [3], which is a crucial aspect of the prehospital work. Noticeable is the finding that SPFS scores remain unchanged: In previous literature, fatigue has also been described as “a conscious sensation rather than a physiological occurrence” [24]. Our findings suggest that subconscious changes in homeostatic control systems already take place before crew members become aware of this and start feeling fatigued.

During a full 5-week cycle, both mean PVT and SPFS increased gradually. Although statistical significance was not reached, this is likely a reflection of the cumulative workload for the crews during that period. As participants were only followed for one 5-week rota cycle, we could not determine when participants recovered and returned to their resting state. However, based on previous literature, one might speculate that longer periods of downtime/leave are needed occasionally to restore the baseline [15].

In the aviation industry, fatigue is regarded as “an expected and ubiquitous aspect of life” [25]. Although fatigue is unavoidable, measures can be taken both on personal- and on service level to avoid the impact of fatigue on performance. Our findings confirm that fatigue is unavoidable in medical crews too. However, based on our findings, several specific measures can be proposed to increase awareness and reduce fatigue.

First, creating a bespoke predictive fatigue calculator, like that of the TFA, could increase fatigue awareness and provide a framework to which relevant mitigating options can be applied. When used in the daily safety brief, it would promote awareness of both individual and crew fatigue states and the teams true ‘fitness to fly’. Second, as the highest SPFS scores were related to car-bound shifts, careful consideration should be given to dispatch crews over long distances when there is a high potential for a stand-down. Further, consideration should be made for doctors to drive the ‘dead legs’ on return from missions or re-positioning for cover to alleviate fatigue on paramedics who do the blue light response driving. Third, alternative shift patterns may be considered; Unlike in the general EMS population, evidence finds 24-h shifts within the HEMS realm provide more rest opportunities and ultimately reduce risk by controlling working hours, suggesting that clinicians become more rested over the length of the shift with lower fatigue ratings on 24-h shifts [12, 13, 26–28]. Finally, monitoring of fatigue by incorporating validated tools in daily practice during shifts may help to increase awareness and to mitigate risk associated with fatigue. This can be applied either as a broad tool or targeted to specific high-risk elements of any rota pattern.

### Limitations

This study has several limitations. First, participants were selected by non-probability convenience sampling. Although this method allows exploration of whether a particular trait or characteristic exists in the sample itself (internal validity), it is acknowledged that sampling bias may occur, resulting in higher-than-normal response rates [29]. Second, our sample size was relatively small due to the low number of full-time employees, which may have attributed to some of the trends observed not being statistically significant. However, this is likely a reflection of the reality in many HEMS services. Third, findings may not be generalizable across the whole workforce as line-share and emeritus staff, who are also engaged in secondary employment, were not included. However, results are likely applicable to them as well; Previous research [26] has demonstrated that those with outside employment attending 12-h night shifts were 90% more likely to attend sleepless than full-time staff. Finally, although trends in both objective and subjective fatigue were observed, due to our study design we can draw no conclusions on causality.

## Conclusion

An overall trend towards a decline in psychomotor vigilance and an increase in self-reported tiredness was found for HEMS crew over a 5-week shift cycle. This highlights the risk of attentional deficits and the prolonged effect of low-level exhaustion placing the organisation at risk of burnout. These results merit further investigation to understand the causality of the trend to mitigate this. The large interindividual variation amongst participants suggests a range of mitigating measures may be necessary to maximise performance. The study provides valuable insight for local decision making to maintain optimum performance of our crews whilst increasing the safety and well-being of service users and clinicians alike. The implementation of informed mitigating measures and promotion of dialogue around working practices could preserve the longevity of these highly functioning teams.

## Data Availability

The datasets used and/or analysed during the current study are available from the corresponding author on reasonable request.

## Abbreviations

HEMS: Helicopter Emergency medical Service
PVT: Psychomotor vigilence testing
SPFS: Samn-Perelli Fatigue scale
TFA: Transport Fatigue Assessment Shift log
